# Effect of an educational intervention based on self-efficacy theory and health literacy skills on preventive behaviors of urinary tract infection in pregnant women: A quasi-experimental study

**DOI:** 10.1371/journal.pone.0306558

**Published:** 2024-08-13

**Authors:** Seyedeh Belin Tavakoly Sany, Vajieh Eslami, Elaheh lael-Monfared, Vahid Ghavami, Nooshin Peyman

**Affiliations:** 1 Social Determinants of Health Research Center, Mashhad University of Medical Sciences, Mashhad, Iran; 2 Faculty of Health, Department of Health, Safety, and Environment, Mashhad University of Medical Sciences, Mashhad, Iran; 3 Faculty of Health, Department of Health Education and Health Promotion, Mashhad University of Medical Sciences, Mashhad, Iran; 4 Department of Health Education and Health Promotion, School of Health, Mashhad University of Medical Sciences, Mashhad, Iran; 5 Faculty of Health Sciences, Department of Epidemiology and Biostatistics, Mashhad University of Medical Sciences, Mashhad, Iran; University of Oulu: Oulun Yliopisto, FINLAND

## Abstract

**Objective:**

The impact of self-efficacy and health literacy skills on pregnant women’s adherence to urinary tract infection (UTI) preventive behaviors is inadequately investigated. Thus, the present study explored whether an educational intervention based on self-efficacy and health literacy skills managed to improve UTI preventive behaviors among pregnant women.

**Methods:**

A quasi-experimental study was conducted from January to July 2021 among pregnant women residing in Mashhad, Iran. To this aim, 110 pregnant women at a gestational age of 12–18 weeks were randomly assigned to a control (n = 55) and an intervention group (n = 55) and completed all questionnaires during the intervention and the 3-month follow-up. The intervention group received the full training program, comprising six 2-hourly training sessions.

**Results:**

Most women were from low-income families (69.1%), were housewives (74.5%) with high school education or lower (63.6%). The theory-based intervention had a significant effect (P < 0·05) on UTI preventive behavior outcomes (i.e., clothing habits, nutrition, urination, health, and sexual behaviors) in the intervention group compared with the control group after intervention, and in their variation from baseline to follow-up in all scores.

**Conclusions:**

An educational intervention based on health literacy skills and self-efficacy could be an effective theory-based intervention to improve UTI preventive behaviors and reduce recurrent UTI and complications.

## 1. Background

Pregnancy is a natural physiological process in a woman’s life, accompanied by physiological and psychological changes. However, maternal comorbidities or unexpected diseases can complicate pregnancy and have adverse effects. Thus, a mother’s health before and during childbirth is very important for children’s health [1,2].

Urinary tract infection (UTI) is a common clinical disease marked by a continuous and active proliferation of bacteria inside the urinary tract [3], and involves the urinary tract, bladder and kidney infections. UTI may be symptomatic or asymptomatic [4] with the latter being of a particular importance due to the absence of any symptoms. Its complications account for about 150 million mortalities annually worldwide [5]. UTI is a common bacterial infection and the second main complication of pregnancy, after anemia. Anatomical and physiological changes of the urinary tracts during pregnancy increase the prevalence of UTI [6]. The prevalence of asymptomatic bacteriuria in the world is 2–15% [7]. In Iran, the prevalence of UTI in pregnant women is 8.7% [8].

In developing countries, pregnant women have a higher rate of UTI than counterparts in developed countries [9]. In a meta-analysis, the overall prevalence of UTI during pregnancy in Iran was estimated at 13%. In different parts of Iran, this rate varied greatly. For example, in Tehran and Arak, it is 2–13%, in Hamadan 10%, and in Torbat Heydarieh, it is reported to be 10% [10]. UTI is among the most widespread and costly medical complications in pregnancy which accounts for 10% of all hospitalizations during pregnancy [11]. As the existing literature shows, UTI in pregnant women begins at the 6^th^ week of pregnancy and reaches its peak in the 22^nd^ - 24^th^ week [2,8].

Besides the high cost of treatment and hospitalization, UTI during pregnancy has many lifelong maternal and fetal complications, including pyelonephritis, preeclampsia, shock, septicemia, anemia, and endometritis. Fetal complications of UTI during pregnancy include birth weight loss, premature birth, respiratory failure, fetal death, mental retardation, and lower intelligence quotient (IQ) [4,9]. The report of the World Health Organization (WHO) on premature birth shows that every year a million infants die due to premature birth [12] and that the probability of preeclampsia in pregnant women with UTI is 1.22 times as high as pregnant women without UTI [13]. Antibiotics are essential to fight UTIs during pregnancy [12], but an excessive use is a global health threat as it can develop antimicrobial resistance and increase the risk of spontaneous abortion and birth defects [14,15]. The consumption of safe antibiotics during pregnancy is limited due to their teratogenic potential [16]. In light of the aforementioned issues, several measures can be taken to prevent UTI during pregnancy, such as adherence to healthy behaviors in sexual activity, the clothing style, eating habits, urinary habits and cleaning, which are all among the predisposing factors for UTI [17,18].

Inadequate knowledge and skills can decrease the motivation to adopt preventive behaviors and can hinder a full prevention [17]. Health literacy skills and self-efficacy are effective factors to prevent infectious diseases [19–21]. In the related literature, health literacy is “a set of reading, listening, analysis and decision-making skills, and the ability to apply these skills in health-related conditions” [22,23]. American Center for Health Care Strategies reported that people with higher health literacy have more chances of using spoken and written information provided by professionals; therefore, they have a better state of health. Health literacy skills improve the acquisition of knowledge about health issues, correct decisions about health, and benefits of healthcare services [24,25]. Problems related to lifestyle changes require a high level of self-confidence. Achieving high self-efficacy, and improving self-efficacy and health literacy is possible through active education [26].

Choosing a behavior change model for health education is the first step to a planning process [18,27]. A prominent educational theory used to predict and describe behavior is the self-efficacy theory, commonly used in behavior changing programs[28]. According to Bandura, there are four main sources of self-efficacy including mastery experiences, vicarious experiences, verbal persuasion, and physiological and affective states” [29]. Self-efficacy is a major prerequisite for behavior change [30]. Individuals with inadequate self-efficacy are less likely to make efforts to show a new healthy behavior or to change the former unhealthy behavior [31].

There is research evidence that self-efficacy is an important psychological construct directly and indirectly affecting disease-controlling health behaviors. Self-efficacy can transform knowledge and information related to health promotion and educational interventions in behavioral performance [32]. Health literacy has been included as a predictor of self-efficacy [32,33]. Although a body of research in Iran shows that women’s awareness of UTI prevention is at a satisfactory level, the prevalence of UTI in pregnant women is still increasing [8,17,34]. It seems that only raising the level of knowledge cannot lead to the prevention of UTI [35–37], and there is a need for recognition of other factors affecting on UTI preventive behaviors [38]. Research evidence shows that self-efficacy and health literacy skills are effective in improving health behaviors. Yet, the relationship between UTI preventive behaviors and health literacy and self-efficacy in pregnant women has not been investigated. Considering the high prevalence of UTI during pregnancy and the serious risks that threaten the mother’s and fetus’ health, the present study aimed to investigate the effect of a health education intervention based on the self-efficacy theory and health literacy skills on pregnant women’s UTI preventive behaviors. The present findings can help decision-makers develop a comprehensive educational program to promote UTI preventive behaviors. Preventive behaviors against UTI helps reduce the excessive use of antibiotics, especially in pregnant women.

## 2. Materials and methods

### 2.1. Participants and sampling

The present quasi-experimental study was conducted from January 2021 to July 2021, among pregnant women living in Mashhad, Iran. In this study, four centers were randomly selected from a total number of 10 health centers through a cluster random sampling. The health centers were selected from areas culturally similar to the intervention group. The inclusion criteria were female participants experiencing their first pregnancy, having no UTI at the time of study, gestational age of 12–18, personal consent to take part in study, being literate, age range of 20–45 years, no history of hospitalization in the past three months, not taking antibiotics or drugs that could inhibit the immune system, not having gestational diabetes mellitus (GDM), a renal disease, and hypertension. Participants were excluded if they did not answer all questions, were unwilling to continue with the study, and were absent for more than one session in the training classes. All eligible participants signed an informed letter of consent. To estimate the sample size, Eq 1 was used:

$$\begin{array}{c} m_{\mathrm{repeated}}=R[{\left(1+\frac{1}{\lambda }\right)}^{2}\frac{{\left(z_{1-\alpha /2}+z_{1-\beta }\right)}^{2}}{\Delta_{\mathrm{plan}}^{2}}+\frac{z_{1-\alpha /2}^{2}}{4}]\\ \begin{array}{l} R=[\frac{1+(w-1)\rho_{T}}{w}-\frac{v\rho_{T}^{2}}{\left[1+(v-1)\rho_{T}\right]}]\\ v\geq 0\\ w\geq 1 \end{array} \end{array}$$ Eq 1

*λ*: ratio of sample size in group 2 to group 1

*v*: number of measures before intervention

*w*: number of measures after intervention

*p*_*t*_: correlation coefficient of repeated measures

*Δ*_*plan*_: standardized expected effect size

Due to the lack of data required to estimate the sample size in the existing literature (e.g., the absence of standard deviation of scores after the intervention in the intervention group), the information about control group in the study by Tehrani et al. [26] was used. The equality of variance of two groups was assumed. Cohen’s standard effect size was 0.56. The first type error was 0.05 and the test power was 80%. *λ*, v, w and p_t._ were, respectively, 1, 1, 2 and 0. 5. The estimated sample size, with an attrition rate of 10%, for each group, was 55. The participants were randomly assigned to the intervention and control groups.

#### Measures

In the present study, the data collection was done based on a test of functional health literacy in adults [28,30], and the general self-efficacy questionnaire [39]. Also, a researcher-made questionnaire was developed to measure UTI preventive behaviors. This questionnaire included demographic information (occupation, age, education, husband’s education and occupation, body mass index (BMI), vomiting during pregnancy, and income) as well as the five domains of UTI prevention behaviors. The questionnaires were completed before, immediately after and three months after the educational intervention in the health centers. All participants were informed about the purpose of study and their demographic information was recorded. Having signed an informed letter of consent, the participants completed the questionnaire of UTI prevention behaviors, test of functional health literacy in adults (TOFHLA) and Schwarzer’s self-efficacy scale.

#### General self-efficacy questionnaire (GSE)

Schwarzer’s general self-efficacy questionnaire was used to measure the participants’ self-efficacy. This scale contained 17 questions rated on a 4-point Likert scale ranging from strongly disagree to strongly agree. It was scored from 17 to 85, and a high score showed stronger self-efficacy. Three aspects of behavior, including the desire to initiate the behavior, resistance to barriers, and efforts to complete the task were measured using this test (e.g., “I am a self-reliant person.”, and “I avoid facing difficulties.”) (S1 Table). The reliability of scale was estimated at 0.84 in the study by Woodruff and Kashman, and 0.83 in the study by Asgharanjad, and Ahmadi Qutb Al-Dini [27,39,40].

#### Test of functional health literacy in adults (TOFHLA)

This questionnaire consisted of two sections, calculations and reading comprehension. The calculations section assessed one’s ability to understand the doctor’s and health educators’ advice. This section required certain calculations, and the score could range between 0 and 50. The reading comprehension section assessed one’s ability to read and comprehend three passages entitled as instructions on preparing for imaging of the upper gastrointestinal tract, the patient responsibilities and rights about the standard hospital consent form and insurance forms. This score ranged from 0 to 50. Thus, the overall health literacy score obtained from these two sections could range between 0 and 100. There were three levels of interpretation of scores: insufficient (0–59), borderline (60–74) and sufficient (75–100) [31]. The validity and reliability of this questionnaire in Iran were measured by Raisi et al. The reliability was estimated at 0.79 for the calculations section and 0.88 for the reading comprehension section. Its content validity ratio (CVR) was higher than 0.56. and the content validity index (CVI) was estimated at 0.79 [28–30].

#### Urinary tract infection preventive behaviors questionnaire

In this study, a researcher-made questionnaire was used to measure UTI preventive behaviors in pregnant women. This questionnaire includes demographic information and five dimensions of UTI preventive behaviors, including 25 questions on clothing style (4 questions), eating habits (6 questions), urinary habits (2 questions), cleanliness (7 questions) and sexual behavioral habits (6 questions) (S2 Table). In this instrument, the questions were rated based on a Likert scale ranging from never (0) to always (4), with a minimum score of 25 and a maximum score of 100. To check the content validity of the researcher-made questionnaire, it was provided to six eminent professors of health promotion, two distinguished professors of reproductive health, two gynecologists and five midwifery experts. Thus, the content validity (CVR) was measured and substantiated. For the overall instrument, the CVR was estimated at 0.94. Having made the suggested revisions, the content validity index (CVI) for all scales was increased to 0.94. To check internal consistency, Cronbach’s alpha test was used, and the estimated value was 0.72. Also, to check the reliability, a test-retest method was used for 20 pregnant mothers at a time interval of two weeks, based on which the intra-cluster correlation coefficient (ICC) was estimated at 0.97, indicating an acceptable reliability [41].

### 2.2. Intervention

**Fig 1:**
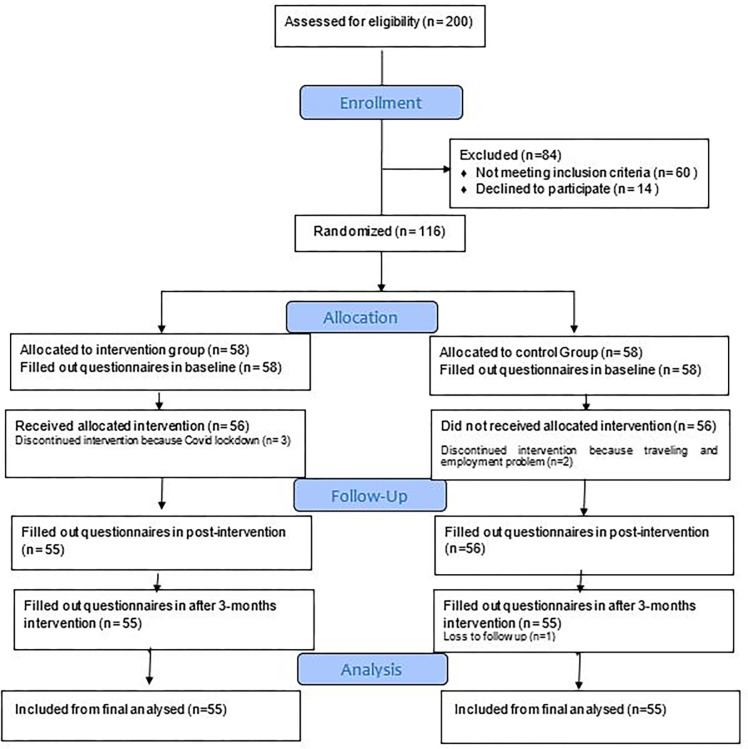
Flow of participants through each stage of the program.

The present quasi-experimental study involved two groups, an intervention, and a control. The intervention was made from January 2021 to July 2021 based on a consort checklist and the Template for Intervention Description and Replication checklist (TIDieR) (Table 1) [42]. Four health centers were selected randomly from a list of centers, and were assigned to the intervention group (n = 2) and the control (n = 2). Then, a list was made of pregnant women’s names based on their demographic information and health history, and a number was assigned to each using a table of random numbers. Two hundred pregnant women were randomly selected, of whom 84 women were not included in the intervention because they did not meet the inclusion criteria. Six women failed to attend the training or follow-up because of travelling, COVID-19 lock-down, and work-related problems. Finally, 110 women completed all stages of study (before intervention, immediately after intervention, and three months after intervention) (Fig 1).

**Table 1:** Intervention description based on the TIDieR checklist.

Items	Descriptions
**BRIEF NAME**	Educational intervention-based health literacy skills and self-efficacy theory to improve UTI preventive behaviors
**WHY** *(Rationale of treatment)*.	Low level of self-efficacy and health literacy skills are correlated with the risk of maternal and fetal mortality and morbidity during pregnancy. Less is known about the effect of educational interventions based on the self-efficacy theory and health literacy skills on UTI preventive behaviors during pregnancy. Therefore, an educational intervention-based health literacy and self-efficacy skills is recommended.
**WHAT** *(Material)*	This intervention was implemented based on Bandura’s self-efficacy strategies, and the health literacy program in practice to improve four areas that are very important in practice, including better oral and written communication, empowerment, and improved support systems.
**WHAT** *(Procedures)*	The intervention group received counselling support and intervention program based on six training sessions with a focus on the key structures of Bandura’s self-efficacy theory and the health literacy program in practice. The control group did not receive any intervention.
**WHO PROVIDED** *(Profession*, *expertise*, *background*, *specific training*)	A qualified health education and promotion specialist and women specialist conducted the training courses. Training sessions were held face to face as well as online. Consulting support was provided through social media and phone contact.
**WHERE** *(Infrastructure and relevant features)*	Training sessions were held in four public healthcare centers in Mashhad, Iran using educational pamphlets, focal group discussion, consulting support, teach-back video, and review action planning reminder cards
**WHEN and HOW MUCH** *(Number of sessions*, *duration)*	six two-hour sessions, every 7 days, for participants in intervention group
**MODIFICATIONS**	No modifications occurred in the intervention or control groups during intervention
**HOW WELL**: *planned (Adherence and procedure to maintain it)*	All eligible participants adhered to the protocol intervention, and only three did not attend the follow-up because of the work-related issue, travelling, and pregnancy.

The educational intervention was conducted for the intervention group. All women underwent a training program of six two-hour sessions every 7 days. From two centers of the control group, 55 pregnant women with similar conditions were randomly selected and considered as the control group, and the educational content was provided to them after the completion of the intervention. Due to the COVID-19 pandemic, the sensitivity of pregnant mothers’ condition and the health protocols against face-to-face group meetings, four training sessions were held face to face, and the remaining sessions were held online on WhatsApp as the mothers requested. In this study, different oral and combined methods (e.g., lectures with Q&As, brainstorming, group discussions, poster presentation and pamphlets) were used along with online sources (e.g., telephone and social networks to share videos, photos and group discussions in real-time class held in audio-only mode) (Table 1). The intervention training program was designed based on Bandura’s self-efficacy theory [27] and health literacy skills (spoken communication, promotion and written communication, empowerment, improvement of support systems) [40] (Table 2).

**Table 2:** Educational intervention-based health literacy and self-efficacy skills to improve UTI preventive behavior.

Education program	Actual Intervention Procedure	Material/methods
**Self-efficacy theory**	To change mother’s ability and self-confidence to modify UTI prevention behaviors despite conflicting situations	lecture, group discussion, barrier/benefit assessment,
Performance accomplishments	Complex tasks were broken into smaller and simpler activities and smaller goals were set. Every time we achieve small goals, we naturally encourage and praise ourselves. Getting these rewards increases interest in the desired activities and makes these activities more continuous. Then, the participants were requested to share their successful experience with others.	
Vicarious Experience	UTI prevention behaviors were explained to participants, and they were asked to discuss the achievements made in practice. This problem strengthened mothers’ belief in the preventability of UTI and the belief that if others have been able to perform the desired behavior, they can succeed too. Participants talked about their achievements and what they did to show UTI preventive behaviors and explained the practical solutions to achieve it.	
Verbal Persuasion	The mothers who managed to show appropriate preventive behaviors were given positive feedback and were encouraged. The possible barriers for mother who failed in carrying out the assigned activities were discussed and practical solutions were provided to overcome these barriers.	
Physiological States	Mothers’ perception of their capabilities and self-monitoring to perform certain behaviors improved. Positive beliefs about UTI preventive behaviors were promoted through explaining the benefits of the preventive behaviors and their effect on the fetus’ and mother’s health. The complications of UTI were explained to involve mother’s feelings because these feelings could lead to the persistency of activities in participants.
**Health literacy skills**	To improve oral communication, written communication, to empower and support systems,	Group discussion
	self-monitoring	Lecture, slides, Reminder card, pamphlet
Oral Communication	Welcoming with a smile and creating a friendly environment, establishing appropriate eye contact, holding face-to-face meetings, using simple language and non-medical terms and using colloquial words, limiting prioritization training content to 3–5 key points, repeating key points, encouraging learners to ask their questions and engaging them in the discussion, asking for feedback	
Written Communication	Using simple and short sentences, developing educational content based on mother’s education level, using fewer specialized terms, defining each word as clearly as possible and using fewer multi-part words, bolding key words, using images and graphics to increase the comprehensibility of message	
Empowerment	Encouraging learners to participate in group discussion and giving appropriate feedback, asking to prepare homework according to the specific goals	
Support systems	Taking time to evaluate problems in performing UTI preventive behaviors, creating a supportive and reliable environment to explain the objectives of intervention clearly and simply, providing easy access to the course trainer (if mothers needed), allowing them to mention any dissatisfaction, receiving further information through phone contact and social media.	
**Preventive behaviors**	Increasing mother’s knowledge and awareness of UTI preventive behaviors, incorporating key concepts to improve woman’s knowledge based on symptoms of UTI, diagnostic measures, complications of UTI, causes of UTI and its treatment, risk factors for UTI, behaviors to prevent UTI habits and hygiene	lecture, slides, teach-back video

In this educational program, according to the participants’ age and literacy level and the objectives of the educational program, there were three cognitive, attitudinal, and functional domains to address, for which visual and auditory media were used such as educational slides, overhead projectors, whiteboards, and pamphlets in this program. Face-to-face training and phone-mediated training were used along with video, photo and voice records in non-face-to-face training. Trainings were conducted by a health education specialist and a gynecologist. During the study, the control group did not receive any special training from the researcher, and after the completion of the intervention, the training was provided as e-learning to the control group. Questionnaires in both groups were completed once before the intervention, and twice more immediately after and three months after the educational intervention. This was done face to face in the first session and online via sharing the questionnaire link in the group to complete.

### 2.3. Data statistical analysis

Having collected the data, to analyze the descriptive data, the questionnaires were coded and punched into SPSS21. After a careful checking and ensuring of the accuracy of data entry, descriptive statistics of the central tendency and variability indices, such as the mean and standard deviation of values related to the interval variables, and the distribution of frequency and percentage of non-parametric variables were used. To check the normality of distribution of interval variables in the treatment and control groups, Kolmogorov-Smirnov test was used. As the results showed, appropriate parametric tests were used for interval variables and appropriate non-parametric tests were used for non-interval variables. To test the relationship between interval variables, Spearman and Pearson correlation coefficients were used according to the abnormal distribution of data. Mann-Whitney U-test, and Kruskal-Wallis tests were used to test the relationship between interval and non-interval variables according to the number of classes of qualitative variables. Chi-square test was used to explore the relationship between non-interval variables. To compare the two groups before, immediately after and 3 months after intervention in terms of interval variables, repeated measure analysis of variance was used. Friedman’s test was used for non-interval variables. The significance level in all tests was 0.05 and SPSS 21 was used to describe and analyze the data.

#### Ethics approval and consent to participate

The study protocol was approved by the Ethics Committee of Mashhad University of Medical Sciences (#IR.MUMS.REC.1398.268) after obtaining the required permit for the research. The participants provided a written informed consent and were assured of confidentiality of data. All procedures performed in studies involving human participants were in accordance with the ethical standards of the institutional research committee with the 1964 Helsinki declaration.

## 3. Results

Before intervention, there were no significant differences (P>0.05) between the intervention and control groups in terms of demographic characteristics (i.e., age, gestational age, education, income, employment status, BMI, history of pre-pregnancy UTI, and vomiting during pregnancy). In this sense, the variables were homogeneous in both groups. The mean (±SD) of age, gestational age, and BMI were 24.80 (±4.92), 13.69 (± 3.82) and 24.93 (±3.18), respectively. Most eligible women were housekeepers (74.5%), low-income families (69.1%) with high school diploma or below (63.6%) (Table 3).

**Table 3:** Participants’ socio-demographic characteristics.

Variables		Control group (*n* = 55)	Intervention group (*n* = 55)	*Statistical Test Result
**Age,** (years), *mean ± SD*	Range: 18–42	24.09*±4*.*78*	25.51*±5*.*07*	^**α**^0.13
**Gestational age**, *(weeks)*, *mean ± SD*	Range: 6–31	13.82*±3*.*74*	13.56*±3*.*91*	^**α**^0.72
**BM**I**,*) kg/m*^2^),*mean ± SD*	Range: 18.07–9.23	25.40*±3*.*30*	24.47*±3*.*07*	^α^ 0.127
**Women’s Education level**, *(n)%*	Diploma/under diploma	23(41.8)	26(47.3)	^**β**^0.56
	Higher education	32(58.2)	29(52.7)	
**Spouse’s Education level**, *n (%)*	Diploma/Under diploma	35(63.6)	26(47.3)	^**β**^ 0.08
	Higher Education	20(36.4)	29(52.7)	
**Women’s Employment Status**, *(n)%*	Housewife	41(74.5)	46(83.6)	^**β**^ 0.24
	Employed	14(25.5)	9(16.4)	
**Spouse’s Employment Status**, *(n)%*	Employee	10(18.2)	9(16.4)	**β** 0.18
	Freelance	20(36.4)	29(52.7)	
	Worker	16(29.1)	14(25.5)	
	Unemployed	9(16.4)	3(5.5)	
**Family Income**, *(n)%*	Low	38(69.1)	41(74.5)	**β** 0.52
	Moderate	17(30.9)	14(25.5)	
**Vomiting in Pregnancy**, *(n)%*	Yes	31(56.4)	33(60)	**β** 0.69
	No	24(43.6)	22(40)	
**History of pre-pregnancy UTI**, *(n)%*	Yes	28(50.9)	20(36.4)	**β** 0.12
	No	27(49.1)	35(63.6)	

*Significant change between intervention and control groups at the 0.05 level; ^α^ Independent ^t^-test; ^β^ chi-square

** Body Mass Index (BMI) was categorized as obese (> 30), overweight (25–29.9), underweight (< 18.5 kg/m2), and normal (18.5–24.9), ±, Showing mean score (standard division); n, number of women; %: percent of total participants.

At the baseline, all UTI preventive behavioral constructs, total preventive behaviors, and self-efficacy were homogeneous in both groups. The results related to UTI preventive behaviors showed a significant improvement (P < 0.05) in all constructs (clothing habits, nutrition, urination, health, and sexual behaviors) in the intervention group at the follow-up, and in all scores changing from baseline to the follow-up. The results of testing self-efficacy showed a significant change (P < 0.05) in the intervention group compared to the control group in the follow-up, and in changes from the baseline to follow-up (Table 4).

**Table 4:** UTI preventive behaviors, self-efficacy, and health literacy from baseline to follow-up in control and intervention groups control and intervention groups.

Model’s Constructs	Pre-intervention					Post-intervention					3- month follow-up					From pre v. follow-up	
	Control		Intervention		*p-value	Control		Intervention		*p- value	Control		Intervention		*p-value	Control	Intervention
	*Mean*	*SD*	*Mean*	*SD*		*Mean*	*SD*	*Mean*	*SD*		*Mean*	*SD*	*Mean*	*SD*		** *p-value*	** *P-value*
**Eating Habits**	61.9	9.5	63.1	7.5	0.411	63.0	9.1	81.3	7.6	<0.001	60.0	8.5	75.9	7.8	<0.001	0.1	<0.001
**Urinary Habits**	66.9	18.9	66.3	16.8	0.894	66.2	18.9	90.0	10.0	<0.001	65.8	18.9	90.0	10.0	<0.001	0.87	<0.001
**Sexual Habits**	66.1	13.9	61.6	11.7	0.071	68.1	12.8	84.7	6.6	<0.001	68.5	12.1	81.3	8.3	<0.001	0.07	<0.001
**Way of Cleaning**	64.4	10.7	64.6	8.9	0.586	64.8	10.6	86.2	7.3	<0.001	65.5	11.0	82.0	6.9	<0.001	0.052	<0.001
**Clothing Habits**	62.84	12.1	66.7	14.4	0.151	63.1	11.2	86.1	8.9	<0.001	68.1	11.6	85.6	7.5	<0.001	<0.001	<0.001
**Preventive behaviors**	64.1	7.04	64.0	6.2	0.909	65.1	6.3	85.0	4.5	<0.001	65.4	6.3	81.6	4.0	<0.001	0.082	<0.001
**Health literacy**	56.3	16.6	55.8	15.9	0.866	56.9	16.1	59.9	15.3	0.626	56.2	15.9	59.3	15.4	0.624	0.08	<0.001
**Self-Efficacy**	55.8	13.7	55.8	12.2	0.801	56.6	13.1	62.1	11.1	0.032	56.5`	13.0	61.9	12.1	0.045	0.071	<0.001

** significant change from pre-intervention to 3-month follow-up based on the Mann–Whitney U test; *significant change between control and intervention groups based on the independent t-test; n, number of women: SD, standard division.

The mean health literacy score immediately after the intervention and three months later was significantly different in the intervention group. The mean score of health literacy was significantly different within the intervention group (p < 0.001). There was no significant difference (P > 0.05) in the change of UTI preventive behavior constructs, total preventive behaviors, self-efficacy, and self-efficacy in the control group at the follow-up (Table 4). The results presented in S3 Table showed that the incidence of UTI three months after the intervention in the control group was 25.4%. The control group significantly had more cases with UTI than the intervention group.

In this section, a generalized estimating equation (*GEE*) model was used to simultaneously measure the effect of intervention, time, self-efficacy, and health literacy on UTI preventive behaviors. The results of the GEE model were in line with the bivariate analysis that showed significant interactions between groups and time. S4 and S5 Tables showed the impact of intervention based on health literacy and self-efficacy on improving UTI preventive behaviors in different groups and times. Changes in UTI preventive behavior score within the intervention group were significantly higher than the control (P = 0 <0.001), and UTI preventive behaviors were increased considerably across time in the baseline through follow-up among participants in the intervention group compared with the control (P = 0 <0.001). As the results showed, changes in self-efficacy (p = 0.043) and health literacy (p = 0.042) were significantly associated with UTI preventive behaviors.

## 4. Discussion

Due to the prevalence of UTI in pregnant women, UTI is considered a major concern in public health public health [41,43,44]. The present finding suggests that conducting an educational intervention based on the self-efficacy theory and health literacy skills among pregnant women is an effective intervention to control and prevent UTI because women in the intervention group represented a lower risk of UTI and better preventive behaviors compared with participants in the control group.

The present findings showed a significant increase in the level of preventive behaviors in the intervention group. Before the intervention, there was no significant difference between the intervention and control groups. However, after the educational intervention, this difference was statistically significant. The present study showed that the educational intervention based self-efficacy and health literacy skills and the use of educational strategies and programs such as the mastery of alternative behavior and verbal persuasion, educational methods such as goal-setting and role-play were effective in improving preventive behaviors. As the present findings showed, the use of the self-efficacy theory can be effective in improving perceived self-efficacy in individuals. It seems that women with adequate self-efficacy and health literacy may well find and use health information and engage in their care [18,45].

In this study, pregnant women in the intervention group showed a significant change in the mean score of self-efficacy after the intervention. All women learned how to break complex tasks into smaller and simpler activities and set realistic goals to modify their action and commitment to conduct UTI preventive behaviors despite conflicting conditions. Likewise, the present researchers tried to improve mother’s self-confidence and self-monitoring to perform certain behaviors. The existing literature shows that individuals with low self-efficacy use fewer resources of health information and health literacy to improve their health or change the habitual behaviors [33]. The findings reported by Osborn et al., 2011 are consistent with the present findings, as individuals with higher perceived self-efficacy had a better understanding of their health state and used health information and health literacy to improve their health and show self-care behaviors [34,46]. The results of the present study are in line with a body of research by Hejazi et al. [47], Abdullahi et al. [48], which showed a significant effect of self-efficacy on adopting, initiating, and maintaining healthy behavior. They found that self-efficacy acted as a moderator to link healthy behaviors with motivation and knowledge [49,50].

The results of the present study showed statistically significant differences in the change of health literacy skills in the intervention group at 3-months follow-up, and in changes from baseline to follow-up in all scores. Health literacy is the main skill to influence one’s ability to use health information, make well-informed decisions, and maintain good health [38,40,42,43]. Before the intervention, women had difficulty finding and comprehending health information and healthcare services to make well-health decisions. Likewise, a significant improvement in the health literacy score was found in the intervention group in post-intervention and follow-up. This could be due to the improved women’s willingness and ability to involve in behaviors and care that improve their health. In the present study, a supportive and reliable environment was created to address health information and measures that contribute to a higher stage of well-health decisions and commitments among pregnant women to modify their UTI preventive behaviors [4,6]. Therefore, it is essential to promote health literacy skills in community, as high health literacy is associated with better health outcomes among patients.

Therefore, it is necessary to plan and implement model-based educational programs based on the self-efficacy theory and health literacy skills to increase pregnant women’s self-efficacy and health literacy. The results of the present study are in line with a body of research. In a descriptive study conducted on 140 pregnant women in Zahedan based on the Health Belief Model (HBM), Rahimi et al. showed that self-efficacy was the strongest predictor of preventive behaviors against UTI. It seems that the reasons for the greater effect of self-efficacy are women’s self-confidence and awareness of the effect of simple behaviors and measures to control UTI [45,46]. In a quasi-experimental study conducted on 60 mothers to children under 6 years of age, Hashemiparast et al. showed the mean self-efficacy score was increased in the intervention group after the intervention. In this study, self-efficacy implied confidence in one’s ability to perform UTI preventive behaviors [47]. Eshghi Mutlaq et al. (2016) found that their educational intervention had a significant effect on improving self-care behaviors in mothers with prediabetes during pregnancy, who felt more self-efficacious and capable of understanding their positive state of health. They also showed showed diabetes self-care behaviors in their daily life [48]. In line with the present study, Ebrahimipour et al. (1994) conducted some research on the effect of an educational intervention based on the self-efficacy theory on the adoption of HIV-AIDS preventive behaviors in high-risk women. This study showed that the educational intervention based on self-efficacy strategies could significantly increase the adoption of self-care behaviors in the intervention group (P<0.001) [49]. Ha et al. also showed that the educational intervention and the use of educational strategies and programs such as mastery of alternative behavior and verbal persuasion, educational methods such as goal-setting and role-play were effective in improving self-care [45]. As the present study showed, the use of this theory proved effective in improving perceived self-efficacy in individuals. The findings emphasized the importance of self-efficacy in preventive behaviors as a suitable educational alternative for UTI self-care and prevention in pregnant women. Therefore, it is necessary to plan and implement model-based educational programs to increase pregnant women’s self-efficacy.

It seems that women with adequate self-efficacy and health literacy may well find and use health information and well engage in their care [18,46,50]. Limited studies exist, investigating the role of self-efficacy and health literacy pandemic conditions influencing awareness and health behaviors among pregnant women. Therefore, further studies need to be conducted on enhancing women’s capability to improve health prevention behaviors toward the different diseases, and focusing on health literacy skills and self-efficacy strategies cause persistent and long-term health behaviors.

The strengths of our findings lie in determining the role of self-efficacy and health literacy using valid instrument among the pregnant women as the groups at risk. Our findings highlighted self-efficacy and health literacy skills as the main modifiable determinants to control and manage unborn child’s health and mother’s health because an adequate level of health literacy and self-efficacy improved individual’s healthy behaviors and health outcomes. Future research on intervention-based health literacy and self-efficacy skills will continue in Iran because this type of training for individuals empowers communities to engage in their self-care, improve the healthy behavior, and can increase valuable health outcome in strengthening healthcare delivery. Therefore, it would be worthwhile to study the modifying health literacy and self-efficacy as a long-term measure.

In this study, the data collection instrument was self-reporting, which can cause problems such as recall and distraction. Due to the COVID-19 pandemic, the questionnaires were not filled face to face. Instead, the questionnaire hyperlink was shared with the pregnant women to fill out the questionnaires in their convenience. In this type of questionnaire completion, errors occur more often, and the researcher has no control over the respondents, which reduces the number of visits by pregnant women to the health centers, as well as attendance to face-to-face training sessions.

## Conclusion

Finally, the results of the present study showed that the educational intervention based on the self-efficacy theory and health literacy skills can be effective in improving UTI preventive behaviors. The promotion of UTI preventive behaviors in pregnant women after the intervention showed that holding training sessions based on the self-efficacy theory and health literacy was useful. Such a training can improve preventive behaviors. The results of this research can be used to increase UTI preventive behaviors in all sex and age groups and can reduce recurrent UTI complications. Also, the present findings can help health system managers formulate intervention programs specifically for employees to prevent office infection and increase health indices while maintaining the health of the mother and the fetus. Development of educational programs by managers for health workers aiming to raise the awareness of women visiting health centers can reduce economic and psychological costs imposed on society.

## Supporting information

S1 Table Scherer general self-efficacy questionnaire. (DOC)

S2 Table Distribution of urinary tract infection prevention behaviors. (DOC)

S3 Table UTI ratio in control and intervention groups at follow-up. (DOC)

S4 Table Effectiveness of the intervention on improving the UTI preventive behaviors via self-efficacy in different group and time period. (DOC)

S5 Table Effectiveness of the intervention on improving the UTI preventive behaviors via Health literacy in different group and time period. (DOC)

## Abbreviations

UTI: urinary tract infection
WHO: World Health Organization
