# Supplementary material for: Effect of an educational intervention based on self-efficacy theory and health literacy skills on preventive behaviors of urinary tract infection in pregnant women: A quasi-experimental study
Source: PLoS One. 2024 Aug 13;19(8):e0306558. doi: 10.1371/journal.pone.0306558 (PMC11321562; doi:10.1371/journal.pone.0306558)
Supplement: S1 Table — (DOC) [file pone.0306558.s001.doc]

Supplementary Material

Table S1. Scherer General Self-Efficacy Questionnaire

|  | **Questions** | **Completely disagree** | **Disagree** | **Average** | **Agree** | **Quite agree** |
| --- | --- | --- | --- | --- | --- | --- |
| 1 | When I make plans, I am certain I can make them work. |  |  |  |  |  |
| 2 | One of my problem is that I can not get down to work when I should. |  |  |  |  |  |
| 3 | If I can’t do a job the first time, I keep trying until I can. |  |  |  |  |  |
| 4 | When I set important goals for myself, I rarely achieve them. |  |  |  |  |  |
| 5 | I give up on things before completing them. |  |  |  |  |  |
| 6 | I avoid facing difficulties. |  |  |  |  |  |
| 7 | If something looks too complicated, will not even bother to try it. |  |  |  |  |  |
| 8 | When I have something unpleasant to do, I stick to it until I finish it. |  |  |  |  |  |
| 9 | When I decide to do something new, go right to work on it. |  |  |  |  |  |
| 10 | When trying to learn something new, I soon give up if I am not initially successful. |  |  |  |  |  |
| 11 | When unexpected problems occur, I don’t handle them well. |  |  |  |  |  |
| 12 | I avoid trying to learn new things when they look too difficult for me. |  |  |  |  |  |
| 13 | Failure just makes me try harder. |  |  |  |  |  |
| 14 | I feel insecure about my ability to do things. |  |  |  |  |  |
| 15 | I am a self-reliant person. |  |  |  |  |  |
| 16 | I give up easily. |  |  |  |  |  |
| 17 | I do not seem capable of dealing with most problems that come up in life. |  |  |  |  |  |
