# Supplementary material for: Effect of an educational intervention based on self-efficacy theory and health literacy skills on preventive behaviors of urinary tract infection in pregnant women: A quasi-experimental study
Source: PLoS One. 2024 Aug 13;19(8):e0306558. doi: 10.1371/journal.pone.0306558 (PMC11321562; doi:10.1371/journal.pone.0306558)
Supplement: S2 Table — (DOC) [file pone.0306558.s002.doc]

Supplementary Material

Table S2. Distribution of urinary tract infection prevention behaviors

| **Questions** | **Always** | **Sometimes** | **Rarely** | **Never** |
| --- | --- | --- | --- | --- |
| **Clothing Habits** |  |  |  |  |
| I wear loose pants |  |  |  |  |
| I wear cotton underwear |  |  |  |  |
| I change my underwear 3 or more times a week |  |  |  |  |
| I use underwear |  |  |  |  |
| **Nutrition** |  |  |  |  |
| I drink 8 glasses or more of water daily |  |  |  |  |
| I drink 3 or more cups of tea a day |  |  |  |  |
| I drink 1 glass or more of soft drink a day |  |  |  |  |
| I drink 1 cup or more of coffee a day |  |  |  |  |
| I use yogurt and milk daily or one day. |  |  |  |  |
| 1 to 2 times a week I use sour drinks such as (barberry juice or blueberry, etc.) |  |  |  |  |
| **Urination** |  |  |  |  |
| When I feel like urinating, I refrain from urinating |  |  |  |  |
| I urinate about 1 hour after the first feeling of urination |  |  |  |  |
| **Health behaviors** |  |  |  |  |
| After using the toilet, I first clean the urethra and then the anus |  |  |  |  |
| I use the pitcher to purify myself |  |  |  |  |
| After using the toilet, I use a towel to dry myself |  |  |  |  |
| I use paper towels when using public toilets |  |  |  |  |
| I take a standing bath |  |  |  |  |
| I take a bath 3 or more times a week |  |  |  |  |
| I dry my underwear in the sun |  |  |  |  |
| **Sexual behavior** |  |  |  |  |
| I urinate before sex |  |  |  |  |
| I wash my genital area before sex |  |  |  |  |
| I urinate shortly after sex |  |  |  |  |
| I wash my genitals shortly after sex |  |  |  |  |
| My wife washes the genital area before intercourse |  |  |  |  |
| If I have a urinary tract infection, I will not approach for 2 weeks |  |  |  |  |
