# Supplementary material for: Effect of an educational intervention based on self-efficacy theory and health literacy skills on preventive behaviors of urinary tract infection in pregnant women: A quasi-experimental study
Source: PLoS One. 2024 Aug 13;19(8):e0306558. doi: 10.1371/journal.pone.0306558 (PMC11321562; doi:10.1371/journal.pone.0306558)
Supplement: S3 Table — (DOC) [file pone.0306558.s003.doc]

Supplementary Material

Table S3: UTI ratio in control and intervention groups at follow-up

| **Variables** |  | **Control group**  **(*n*=55)** | **Intervention group**  **(*n*=55)** | ***^*^*P-value** |
| --- | --- | --- | --- | --- |
| UTI Ratio, % | NO | 41(74.6) | 52(94.5) | 0.0180 |
|  | Yes | 14(25.4) | 3 (5.5) | 0.001 |

* Testing significant change between control and experimental groups which is significant at the 0.05 level
