# Supplementary material for: Effect of an educational intervention based on self-efficacy theory and health literacy skills on preventive behaviors of urinary tract infection in pregnant women: A quasi-experimental study
Source: PLoS One. 2024 Aug 13;19(8):e0306558. doi: 10.1371/journal.pone.0306558 (PMC11321562; doi:10.1371/journal.pone.0306558)
Supplement: S4 Table — (DOC) [file pone.0306558.s004.doc]

Supplementary Material

Table S4: Effectiveness of the intervention on improving the UTI preventive behaviors via self-efficacy in different group and time period.

| **Variables** | | **The regression coefficient** | **95 % CI** | ***p-value** |
| --- | --- | --- | --- | --- |
| **Group** | Intervention | 16.175 | 14.417-17.932 | 0<0.001 |
|  | Control | 0 | - | - |
| **Time** | Two months after the intervention | 0 | - | - |
|  | Immediately after the intervention | -0.376 | -0.991-0.240 | 0.232 |
| **Self-Efficacy** | | 0.209 | -0.146-0.365 | 0.0403 |
| **Intervention* Immediately after the intervention** | | 3.679 | 2.459-4.899 | 0<0.001 |

CI, confidence interval; *Testing significant effect between groups and period.
