# Supplementary material for: Effect of an educational intervention based on self-efficacy theory and health literacy skills on preventive behaviors of urinary tract infection in pregnant women: A quasi-experimental study
Source: PLoS One. 2024 Aug 13;19(8):e0306558. doi: 10.1371/journal.pone.0306558 (PMC11321562; doi:10.1371/journal.pone.0306558)
Supplement: S5 Table — (DOC) [file pone.0306558.s005.doc]

Supplementary Material

Table S5: Effectiveness of the intervention on improving the UTI preventive behaviors via Health literacy in different group and time period.

| **Variables** | | **The regression coefficient** | **95 % CI** | ***p-value** |
| --- | --- | --- | --- | --- |
| **Group** | Intervention | 15.981 | 14.281-17.681 | 0<001 |
|  | Control | 0 | - | - |
| **Time** | Two months after the intervention | 0 | - | - |
|  | Immediately after the intervention | -0.513 | -1.132-0.106 | 0.104 |
| **Health literacy** | | 0.316 | -0.018-0.450 | 0.042 |
| **Intervention* Immediately after the intervention** | | 3.824 | 2.59-5.057 | 0<001 |

CI, confidence interval; *Testing significant effect between groups and time period;
